# Evaluation of Bone Sialoprotein Coating of Three-Dimensional Printed Calcium Phosphate Scaffolds in a Calvarial Defect Model in Mice

**DOI:** 10.3390/ma11112336

**Published:** 2018-11-21

**Authors:** Andreas Baranowski, Anja Klein, Ulrike Ritz, Hermann Götz, Stefan G. Mattyasovszky, Pol M. Rommens, Alexander Hofmann

**Affiliations:** 1 Department of Orthopaedics and Traumatology, BiomaTiCS Group, University Medical Center, Johannes Gutenberg University, Langenbeckstraße 1, D-55131 Mainz, Germany; Anja.Klein@unimedizin-mainz.de (A.K.); ulrike.ritz@unimedizin-mainz.de (U.R.); Stefan.Mattyasovszky@unimedizin-mainz.de (S.G.M.); pol.rommens@unimedizin-mainz.de (P.M.R.); ahofmann@westpfalz-klinikum.de (A.H.); 2 Platform for Biomaterial Research, Biomatics Group, University Medical Center, Johannes Gutenberg University, Langenbeckstraße 1, D-55131 Mainz, Germany; hgoetz@uni-mainz.de; 3 Department of Traumatology and Orthopaedics 1, Westpfalz-Clinics Kaiserslautern, Hellmut Hartert-Str. 1, D-67655 Kaiserslautern, Germany

**Keywords:** bone sialoprotein, calcium phosphate cements, bioactive coating, 3D printing, mouse calvarial defect model

## Abstract

The bioactive coating of calcium phosphate cement (CPC) is a promising approach to enhance the bone-healing properties of bone substitutes. The purpose of this study was to evaluate whether coating CPCs with bone sialoprotein (BSP) results in increased bone formation. Forty-five female C57BL/6NRj mice with an average age of six weeks were divided into three groups. Either a BSP-coated or an uncoated three-dimensional plotted scaffold was implanted into a drilled 2.7-mm diameter calvarial defect, or the defect was left empty (control group; no CPC). Histological analyses revealed that BSP-coated scaffolds were better integrated into the local bone stock eight weeks after implantation. Bone volume/total volume (BV/TV) ratios and bone thickness at the bone–implant contact were analyzed via micro computed tomography (µCT) after eight weeks. BSP-coated scaffolds and uncoated CPC scaffolds increased bone thickness in comparison to the control (CPC + BSP: 691.1 ± 253.5 µm, CPC: 603.1 ± 164.4 µm, no CPC: 261.7 ± 37.8 µm, *p* < 0.01). Accordingly, BV/TV was enhanced in both scaffold groups (CPC + BSP: 1.3 ± 0.5%, CPC: 0.9 ± 0.5%, no CPC: 0.2 ± 0.3%, *p* < 0.01). The BSP coating showed a tendency towards an increased bone thickness (*p* = 0.18) and BV/TV (*p* = 0.18) in comparison to uncoated CPC scaffolds. However, a significant increase in bone formation through BSP coating was not found.

## 1. Introduction

Autologous bone grafting is still considered the gold standard in the treatment of critical-sized bone defects; it promises bone remodeling after quick graft integration [1,2]. Yet, limited availability and donor site morbidity point out the necessity for alternatives [3,4]. Extensive research with a focus on calcium phosphate cement (CPC) scaffolds as bone substitutes has been carried out in recent years [5]. Their composition is similar to that of mineral bone, and they have shown to be biocompatible, osteoconductive, and bioactive [6]. Since the development of the spreading technique of rapid prototyping (RP) three-dimensional (3D) plotting, the shapes and sizes of scaffolds can be created specifically. Moreover, the internal pore structure can be adapted, which is important for osteoconductivity and allows vascular ingrowth. Thus, open macroporous CPC scaffolds can be created with a compressive strength of 2–5 MPa, which is close to that of human trabecular bone [7,8]. Besides mechanical stability, an osteoconductive matrix, and an osteogenic cell population in the host tissue, Giannoudis et al. demanded in their diamond concept the presence of an osteoinductive stimulus [9]. The surface coating of materials with bioactive molecules is one possible way of adding the osteoinductive element to a scaffold.

Bone sialoprotein (BSP) is a tissue-specific phosphoprotein of the extracellular matrix (ECM) of bone, and contains several functional domains. Its arginylglycylaspartic acid (RGD) section represents a cell adhesion motif and is able to interact with the α_ν_β_3_-receptor (vitronectin receptor) of osteoblasts [10,11]. Thus, it regulates the attraction and differentiation of osteoprogenitor cells and osteoblasts. Coated on titanium, BSP was shown to increase the gene expression level of RUNX2 of primary human osteoblasts (hOBs) [12]. The BSP coating of CPCs increased osteoblastic viability and triggered hOB differentiation and spreading, which became apparent in a larger cell area and a reduced circularity [13]. O’Toole found out that the BSP coating of titanium implants promoted the recruitment of osteoblastic cells and the formation of osteoid tissue in addition to evidence of neovascularization in a rat femoral model in vivo [14]. Previous in vivo studies demonstrated improved bone healing with BSP in critical-sized calvarial defects in rats [15,16]. In a murine BSP gene knockout model (BSP−/−), the repair of a 1-mm diameter femoral drill hole was delayed respective to wild type [17]. The mineralization of femoral bone was delayed, and medullary bone formation was lower in BSP−/− mice in comparison to wild type in a marrow ablation model [18]. Moreover, both the adhesion and chemotactic migration of human umbilical vein endothelial cells (HUVECs) were promoted by BSP in a dose-dependent manner, which highlights the role of BSP in α_ν_β_3_ integrin-mediated angiogenesis [19]. Via its hydroxyapatite binding site, BSP can be bound to hydroxyapatite, and is therefore capable of inducing a hydroxyapatite cluster formation [20,21]. We observed an increased calcium deposition of hOBs after 21 days in medium supplemented with BSP [12]. All of these favorable characteristics highlight the potential of BSP as a surface modifier that could enhance the osseointegration of bone substitutes. Although these BSP properties sound promising, it is still uncertain whether the BSP coating of CPC scaffolds is of benefit to bone ingrowth in vivo. The purpose of our study was to examine whether BSP-coated CPC scaffolds are superior to uncoated scaffolds in terms of bone formation in a critical-size calvarial defect in mice.

## 2. Materials and Methods

### 2.1. Scaffold Preparation and Coating

Three-dimensional printed CPC scaffolds (2.7-mm diameter, 1.8-mm height) with interconnecting pores were purchased from INNOTERE GmbH (Radebeul, Germany). A mixture of eight parts of CPC powder containing a-tricalciumphosphate (a-TCP, 60%), dicalcium phosphate anhydride (DCP, 26%), calcium carbonate (CaCO_3_, 10%) and precipitated hydroxyapatite (HA, 4%) and two parts of carrier liquid were used for printing the scaffolds with a final Ca/P ratio of 1.5. Details concerning scaffold preparation or the printing process can be found in specified publications [8,22]. Prior to use, the scaffolds were solidified by incubating the cement in 0.9% sodium chloride for 28 days followed by intense rinsing in deionized water, drying, and gamma irradiation. Lode et al. proved the cytocompatibility of the scaffolds in 2014 via cell culture experiments performed with human mesenchymal stem cells (hMSCs) [8]. The scaffolds´ specific surface area was 12 m²/g. The ratio of macroporosity reached 50%, with pore sizes up to 0.069 mm^2^ in the transverse plane, and up to 0.173 mm^2^ in the side wall of the scaffolds (with a double-strand design in the z-axis). Scaffold coating was conducted according to a previously described protocol [13]. First, the scaffolds were preincubated for 24 h in Dulbecco’s Modified Eagle Medium (DMEM)/Ham’s F-12 medium (Gibco^®^, Life Technologies, Grand Island, NY, USA) without any supplements at 37 °C to ensure efficient conditioning (ion exchange and binding of serum proteins to the CPC surface). This step was followed by incubation with BSP solution (200 µg/mL; Immundiagnostik, Bensheim, Germany) or PBS (phosphate-buffered saline; Sigma-Aldrich, Steinheim, Germany) under mechanical stirring at 8 °C for 24 h. Thereafter, the scaffolds were washed two times with PBS, dried under laminar air flow, and stored at 8 °C until scaffold implantation. Successful BSP coating with this method and observations of BSP release were performed in in vitro studies in our laboratory [13]. For qualitative coating evaluation, the scaffolds were coated with a fluorescein-coupled BSP solution. A fluorescein conjugation system (Lightning-Link^®^ Fluorescein Conjugation Kit, Innova Biosciences, Cambridge, UK) was used according to the manufacturer’s instructions prior to the coating step with BSP. The scaffolds were rinsed after the coating step with PBS, dried, cut and visualized with the EVOS^®^ microscope (Life Technologies, Carlsbad, CA, USA).

### 2.2. Study Design

This study was approved by the local ethics committee (G 15-1-050). National regulations for the care and use of laboratory animals were respected at all times. Forty-five female C57BL/6NRj mice with an average age of six weeks were divided into three groups (BSP-coated scaffolds, uncoated scaffolds (treatment with PBS), and negative control (NC, no scaffold implantation)). For evaluation of direct ossification, a critically sized calvarial defect model was performed in mice [23]. Five mice were housed per cage (type 2, with filter hood) under light and dark cycles (12 h) with free cage activity and drinking water as well as food ad libitum.

### 2.3. Anaesthesia and Surgical Procedure

**Figure 1:**
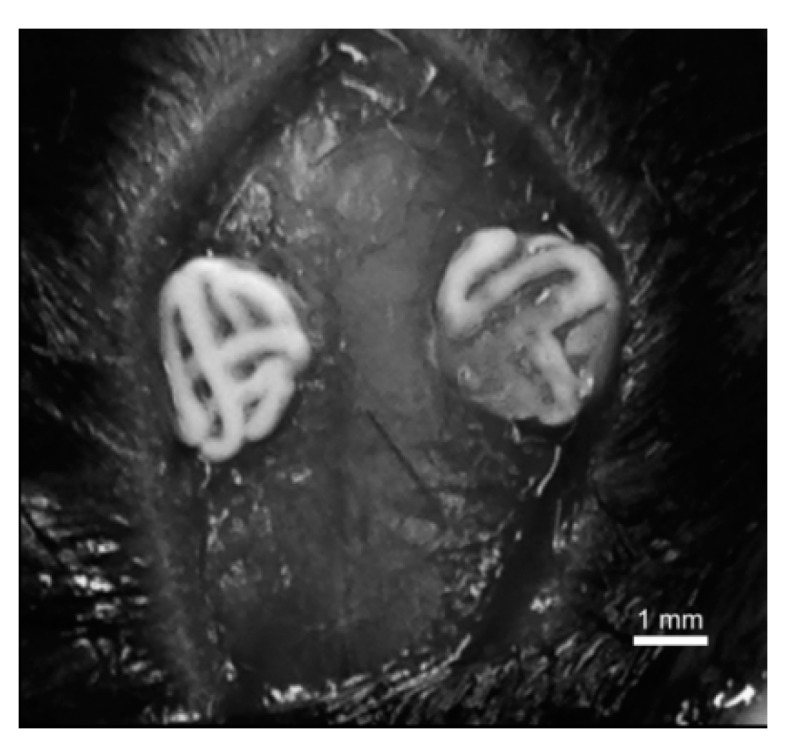
View on freshly implanted calcium phosphate cement (CPC) scaffolds in critical-size defects in mice calvaria before wound closure.

One day prior to the surgical procedure, the drinking water was supplemented with tramadol (1 mg/mL; Ratiopharm, Ulm, Germany). Mice were anaesthetized with an intraperitoneal injection of midazolam (5 mg/kg body weight), medetomidin (500 µg/kg) and fentanyl (50 µg/kg). The animal’s scalps were prepped with Braunol 7.5%, followed by a midsagittal skin incision and exposure of the parietal bone. On each side of the sagittal suture, a bone defect with a 2.7-mm diameter was carefully set with a drill (Hager & Meissinger, Neuss, Germany) using saline water (0.9%; Braun, Melsungen, Germany) for irrigation. Care was taken to avoid lesions of dura mater and superior sagittal sinus. Bone debris was removed under saline irrigation. In the treatment groups, either a BSP-coated scaffold or an uncoated scaffold was implanted in press-fit technique, where applicable (Figure 1). In some cases, press-fit anchoring was not achievable, and the scaffold had some mobility in the bone defect. Defects in the negative control were left empty. The skin was closed with sutures. Drinking water was supplemented with tramadol (1 mg/mL) postoperatively for up to five days.

Mice were killed by CO_2_ inhalation after eight weeks of observation. The fur from the head and neck region was removed, followed by decapitation. Afterwards, the skull was fixed with 4.5% formaldehyde solution (Carl-Roth, Karlsruhe, Germany) for a minimum of three days for further analysis.

### 2.4. Micro Computed Tomography (µCT) Analyses

For µCT measurements, the fixed samples were analyzed with 70 kV and 114 µAmp using the µCT 40 (Scanco Medical, Brüttisellen, Switzerland). The voxel size was set to 30 µm. In the scaffold groups, only defects with direct bone–implant contact (BIC) were included in the statistical data evaluation, as we did not find any bone formation in the absence of direct bone-implant contact (data not shown). For bone thickness examination, this led to a number of evaluable defects of n = 10 (CPC), n = 8 (CPC + BSP), and a size-matched negative control of n = eight defects. In BV/TV measurements, the number of evaluable defects was n = 11 (CPC), n = 9 (CPC + BSP), and a negative control of n = 26 defects. 

### 2.5. Measurements of Bone Thickness at the Bone–Implant Interface

**Figure 2:**
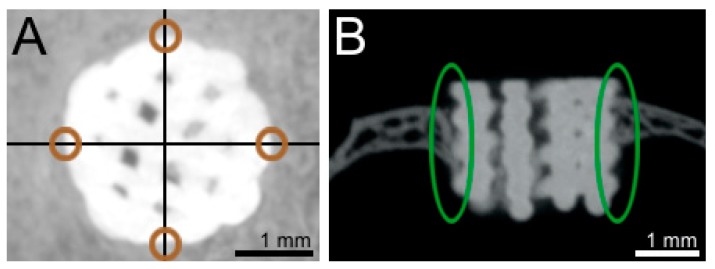
Transverse (**A**) and coronal (**B**) µCT cuts of bone defect and scaffold. Bone thickness (green circles) was measured at four distinct locations (brown circles) per defect.

Bone regeneration at the margins of the defect was analyzed by measurement of bone thickness. Four opposing locations were determined in a horizontal µCT section (Figure 2A). Measurements were carried out in coronal µCT cuts (Figure 2B).

### 2.6. Bone Volume/Total Volume Ratio

Bone volume/total volume (BV/TV) ratios were analyzed using Image J software [24,25] with the BoneJ plugin [26]. For bone volume (BV) evaluation, the grey-value CT data was converted with the help of thresholds into two binaries. Due to different Hounsfield units (scaffold 6695 and bone 3223), a clear separation between bone and scaffold was possible (Figure 2). The first binary consisted of both bone and scaffold data. The second binary included the scaffolds´ data only. Next, the scaffolds´ binary was subtracted from the combined binary. As a result, a new “bone only” binary was gained for further BV/TV analyses. The samples were rearranged using the AlignStacks plugin, followed by analysis with the BoneJ plugin. A cylinder with a diameter of 2.5 mm and a height of 2.2 mm was defined as the region of interest (ROI) and represented the dimensions of the total volume (TV). In order to evaluate bone ingrowth into the scaffolds center, smaller diameters (2 mm, 1.5 mm, 1 mm, and 0.5 mm) were analyzed as well.

### 2.7. Histological Analysis

For histological analysis, the samples were decalcified with a 10% ethylenediaminetetraacetic acid (EDTA, Applichem, Darmstadt, Germany) solution buffered with tris(hydroxymethyl)aminomethane (TRIS, Applichem, Darmstadt, Germany) for three weeks. Then, the tissue was cut close to the ROI, dehydrated, and embedded in paraffin using the SAKURA VIP E150 Tissue Processor (Miles Scientific, Naperville, IL, USA). The embedded samples were cut into 5-µm thick slices. Every fifth section was stained with hematoxylin (Polysciences Europe, Eppelheim, Germany) and eosin (HE; Carl-Roth, Karlsruhe, Germany). For HE staining, samples were deparaffinized with xylene (3 × 10 min; Applichem, Darmstadt, Germany) and rehydrated using a downstream isopropyl alcohol (IPA; Carl-Roth, Karlsruhe, Germany) series (2 × 5 min 100% IPA, 2 × 5 min 96% IPA, 1 × 5 min 70% IPA, 1 × 5 min 50% IPA), which finished in distilled water. Next, the slices were stained with hematoxylin according to Gill (7 min; Polysciences Europe, Eppelheim, Germany) and eosin (3 min; Carl-Roth, Karlsruhe, Germany), followed by an upward IPA series, xylene incubation (3 × 5 min) and coverslipping with Cytoseal^TM^ XYL (Richard-Allan Scientific, San Diego, CA, USA). In addition to HE staining, Masson–Goldner–Trichrom staining (MGT) staining was performed. Besides nucleus and cytoplasm stain, this triple stain highlight different types of connective tissue. Distinctions between bone and connective tissue are apparent by means of different green tones. For MGT staining, the rehydrated samples were stained with hematoxylin according to Weigert (five minutes; Carl-Roth, Karlsruhe, Germany), ponceau-acid fuchsin (10 min; Sigma-Aldrich, Steinheim, Germany), phosphotungstic acid–orange G (10 min; Carl-Roth, Karlsruhe, Germany) and 0.2% light green (10 min; Chroma-Gesellschaft, Köngen, Germany). Differentiation occurred with 1% acetic acid (circa 30 s) between the individual stages.

### 2.8. Immunohistological Analysis

Hydrated samples were incubated with protein kinase K (Dako, Hamburg, Germany) for enzymatic antigen retrieval for 10 min, followed by two washing steps (every step five minutes) in PBS. Endogenous peroxidases were blocked with 3% hydrogen peroxide (Merck, Darmstadt, Germany), followed by several washing steps with PBS and the prevention of unspecific antibody binding with 10% horse serum for 30 min. Incubation with the first antibody was performed over night at 4 °C. Osteopontin (OPN; Abcam, Cambridge, UK), von Willebrand factor (vWF; Life Span Biosciences, Seattle, WA, USA) and platelet endothelial cell adhesion molecule-1 (PECAM-1; Abcam, Cambridge, UK) were assessed. The controls were incubated in PBS with 1% bovine serum albumin (BSA) without antibody (no AB). On the next day, two washing steps with PBS (each five minutes) were performed, followed by incubation with a biotinylated link (Dako, Hamburg, Germany) for 10 min, 5 min of PBS twice, and 10 min of streptavidin–horseradish peroxidase (HRP, Dako, Hamburg, Germany). Next, another two washing steps (five minutes) with PBS followed. Incubation with 3,3′-diaminobenzidine (DAB) substrate–chromogen (Dako, Hamburg, Germany) was controlled microscopically. The conversion from 3,3′-diaminobenzidine (DAB) into a brown-colored precipitate was stopped with distilled water. Finally, the sections were counterstained with hematoxylin according to Gill.

### 2.9. Statistical Analyses

SPSS 23.0 (IBM, Chicago, IL, USA) software was used for statistical analyses. Quantitative results were presented as means ± standard deviation. Data were analyzed with a Welch-ANOVA, followed by Games–Howell post-hoc analysis. A p-value < 0.05 was considered statistically significant. Boxplots were presented with medians and quartiles.

## 3. Results

### 3.1. Qualitative Evaluation of BSP Coating

**Figure 3:**
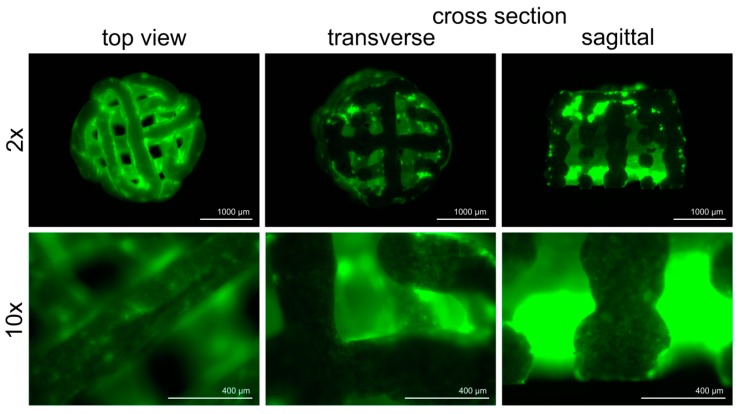
Qualitative evaluation of bone sialoprotein (BSP) coating. BSP was linked with fluorescein prior to the coating procedure. After several washing steps, the remaining BSP (green) was visualized via microscope.

BSP coating was performed with a fluorescein coupled solution to control the success of the coating process. The stability of the coating was indicated by an intensive green fluorescence that persisted after several washing steps with PBS (Figure 3). In order to evaluate the depth of the penetration of the coating solution, the scaffolds were cut vertically and horizontally. As expected, fluorescence intensity was less pronounced in the middle section of the scaffolds.

### 3.2. Bone Thickness

**Figure 4:**
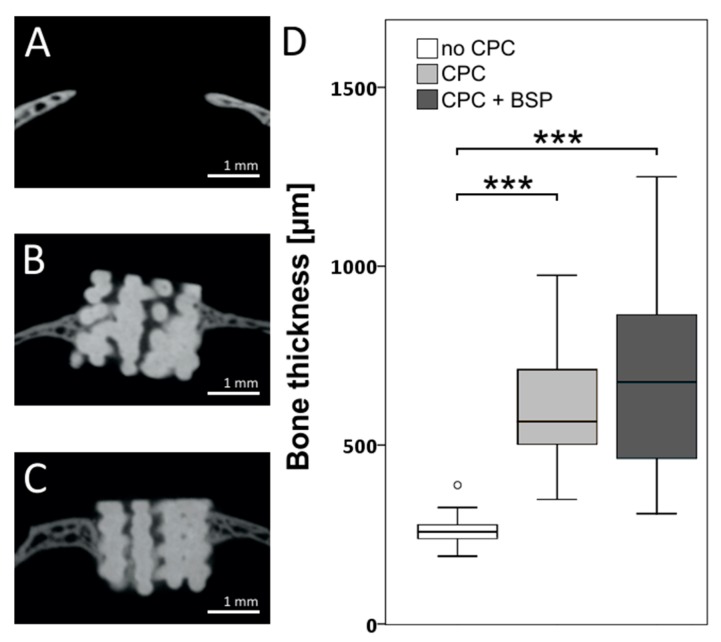
Coronal µCT section of the calvarial defect with inserted scaffold. (**A**) Negative control (no CPC); (**B**) CPC; (**C**): CPC + BSP; (**D**) Boxplots represent bone thickness in µm at the margin of the defect. Statistically significant differences (*p* < 0.001) to the control group are labeled by three asterisks.

New bone formation at the transitional zone between the bone and the defect could be best measured by an assessment of the increasing bone thickness at the margins. Bone thickness at the bone–scaffold interface was not significantly different between the BSP-coated and uncoated scaffolds (BSP: 691.1 ± 253.5 µm, uncoated: 603.1 ± 164.4 µm, p = 0.18). However, both scaffolds demonstrated more than doubled bone thickness in comparison to the bone-defect transition of the control (261.7 ± 37.8 µm, *p* < 0.001) (Figure 4).

### 3.3. Bone Volume/Total Volume (BV/TV)

**Figure 5:**
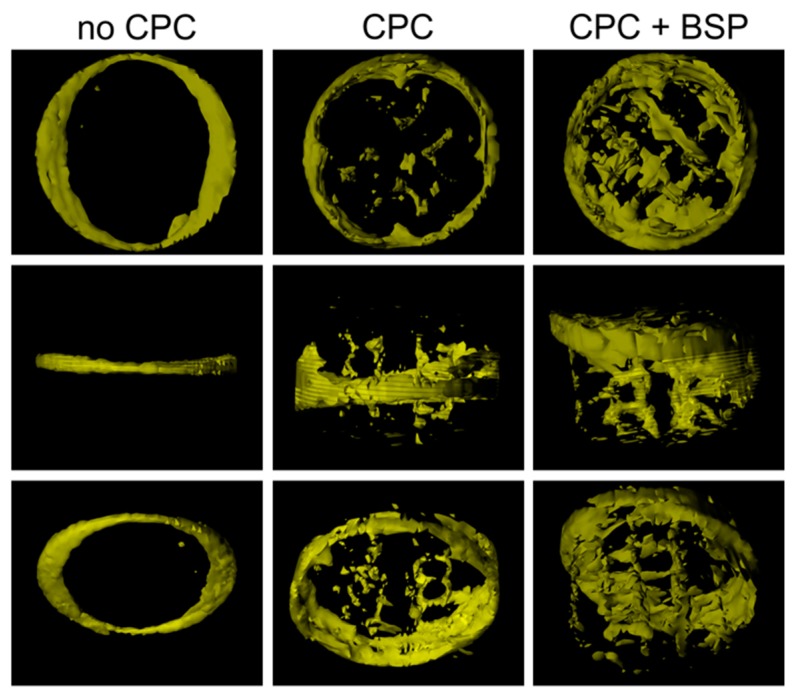
Three-dimensional view of newly formed bone within the region of interest (ROI) from the top view (upper row), the lateral view (middle row), and the oblique view (lower row).

In addition to the measurement of bone thickness at the defect´s margins, calculation of new bone formation within the defect was performed (Figure 5).

**Figure 6:**
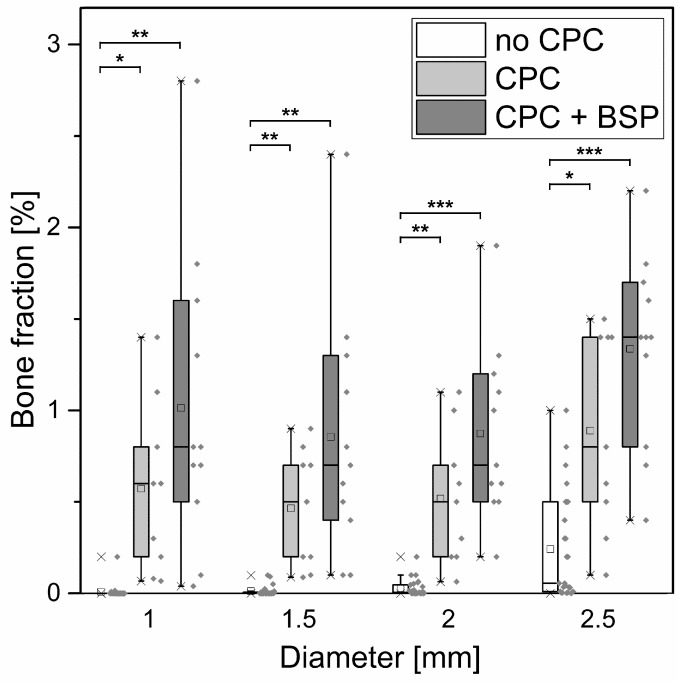
Boxplot of the bone fraction (bone volume (BV)/total volume (TV) ratio) after eight weeks. The core of the scaffold is represented by a virtual cylinder with a diameter of 1.0 mm, whereas the total scaffold is represented by a diameter of 2.5 mm. Therefore, different diameters illustrate bone ingrowth into the different layers of the scaffold. Statistically significant differences are labeled by asterisks (*: *p* < 0.05, **: *p* < 0.01, ***: *p* < 0.001).

Bone formation can be expressed as a bone fraction or bone volume/total volume (BV/TV). In order to be able to make a statement about bone ingrowth into the scaffold, we evaluated not only the total scaffold (diameter of ROI: 2.5 mm, height of 2.2 mm), but also the cylindrical ROIs with the same height but smaller diameters (1.0 mm, 1.5 mm, and 2.0 mm, Figure 6). In the very center of the scaffold (which was defined as a cylinder with a diameter of 1.0 mm and a height of 2.2 mm), we could notice a BV/TV of 0.6 ± 0.5% (CPC) and 1.0 ± 0.8% (CPC + BSP) versus 0.009 ± 0.04% in the control. In the total scaffold, BV/TV was 0.9 ± 0.5% (CPC), 1.3 ± 0.5% (CPC + BSP), and 0.2 ± 0.3% (no CPC), respectively. Although we observed a tendency towards increased bone ingrowth in the outer layers of the scaffolds and a significant difference between both scaffold groups and the control (no CPC), we did not detect a significantly increased bone growth in the BSP-coated scaffolds compared to the uncoated scaffolds (Figure 6). 

### 3.4. Histology

#### 3.4.1. HE Stainings

**Figure 7:**
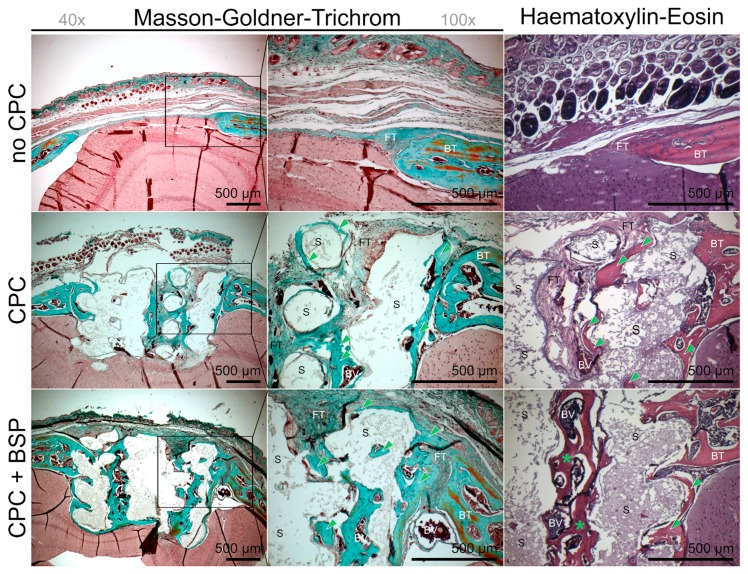
Masson-Goldner-Trichrom (MGT) and hematoxylin-eosin (HE) stainings after eight weeks. New bone growth (green arrows). Trabecular bone structure (green asterisks). BT: calvarial bone tissue at the margins of the defect. BV: blood vessel. FT: fibrous tissue. S: scaffold. BSP-coated scaffolds (CPC + BSP) presented with new bone growth also above and below the scaffold.

Histological assessments were made to complete the picture of scaffold integration and new bone formation. Bone ingrowth was the most pronounced in the BSP-coated scaffolds. The inner rows of the scaffold were tightly covered with bone in the BSP group, whereas the uncoated scaffolds developed new bone predominantly in the peripheral areas. In the negative control, the osseous defect was covered with fibrous tissue only (Figure 7).

#### 3.4.2. Masson-Goldner-Trichrom (MGT) Staining

MGT staining the CPC scaffolds without BSP coating displayed newly formed bone in direct contact with the material, while the pores between the individual strands consisted mainly of connective tissue. In contrast, the BSP coating led not only to bone formation in direct contact with the material, but also inside the pores, as well as above and below the scaffolds (Figure 7). Bone inside the scaffolds’ pores consisted of dense and trabecular parts in the BSP group and resembled the natural calvarial bone, whereas the trabecular parts in the uncoated scaffolds were uncommon. In the negative control, the defect was filled with fibrous tissue, and bone formation could only be observed at the very margins of the defect (Figure 7, no CPC).

#### 3.4.3. Immunohistological Staining

**Figure 8:**
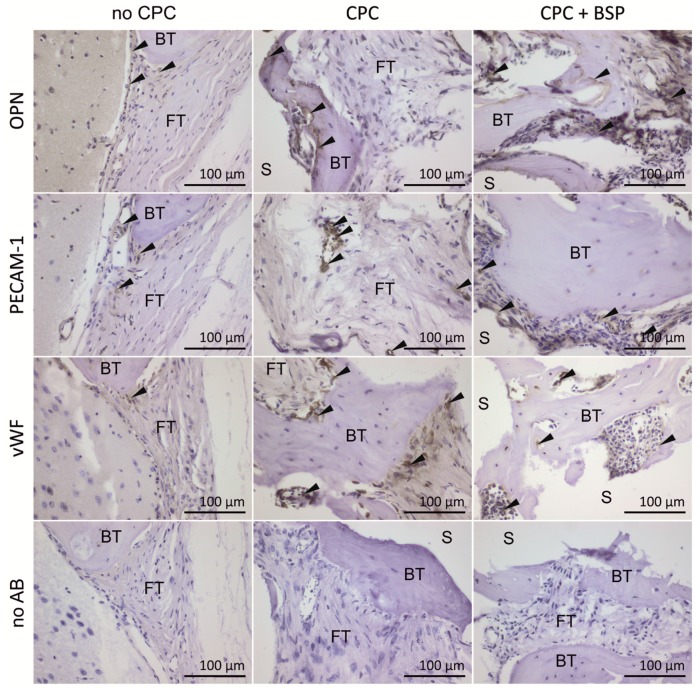
Immunohistological analysis of endothelial and osteoblast cell markers (von Willebrand factor (vWF), platelet endothelial cell adhesion molecule-1 (PECAM-1), and osteopontin (OPN)). Black arrows indicate positive staining (3,3′-diaminobenzidine (DAB) precipitate). Blood vessels in the scaffold’s pores were strongly positive for PECAM-1 and vWF, and were notably pronounced in the BSP group. Cells in contact with newly formed bone were positive for OPN, particularly in scaffolds with BSP coating. There were no stained areas in the sections with no antibody (no AB). FT: fibrous tissue. BT: bone tissue. S: scaffold.

Immunohistological staining was performed for osteopontin (OPN), which plays a pivotal role in the regulation of mineralization within the extracellular matrix (ECM) of bone [27]. OPN is scarcely found in the group without CPC, and only at the margins of the bone defect (Figure 8). In the CPC group, we observed a greater amount of OPN+-cells that were situated around the islets of new bone. OPN+-cells occurred in abundance in the CPC + BSP group, where they enclosed in thick layers not only the newly formed bone, but also the adjoining soft tissue. Stainings of platelet endothelial cell adhesion molecule (PECAM-1 or cluster of differentiation 31 (CD31)) and von Willebrand factor (vWF) were performed to demonstrate the presence of endothelial cells after blood vessel formation. While blood vessels were hardly detectable in the control (no CPC), the histological sections indicated a thorough neovascularization of uncoated and BSP-coated scaffolds (Figure 8).

## 4. Discussion

The integration of bone substitutes into the local bone stock is a complex process. On the protein level, the initial host response is affected by the scaffold’s material and the first layer of proteins on an implant [28,29]. The activation, migration, and differentiation of osteoprogenitor cells are key elements in the synthesis of the extracellular matrix, and are thus essential for osseointegration at the scaffold–bone interface [30]. However, the vascularization of bone substitutes is the mainstay of scaffold remodeling [31]. Particularly in larger scaffolds, vessel penetration assures sufficient oxygen and nutrient supply for cells, even in the scaffold’s center. Therefore, both the improvement of bone formation and angiogenesis are of main interest. BSP coating could fill this gap, as BSP is able to induce hydroxyapatite cluster formation and attract both osteoblasts as well as endothelial cells via their α_ν_β_3_-receptor [19,20,21]. A coating concentration of 200 µg/mL has proven to be advantageous for cell viability and the gene expression of primary human osteoblasts (hOBs) in our in vitro studies [12,13]. Thus, this concentration was chosen for our in vivo model. In our previous works, we were able to show that after an initial burst, CPC scaffolds were capable of binding and retaining BSP via physisorption. During the first 48 h after coating, slow release kinetics with a cumulative release of 0.8–28 µg were observed. This release pattern was similar to that of BSA (bovine serum albumin)-coated CPCs [32,33]. Our hypothesis was that the BSP coating of CPC scaffolds might lead to an increased formation of bone in comparison to untreated scaffolds in vivo. Based on measurements of bone thickness and the BV/TV ratio, we provide sufficient evidence that BSP-coated CPC scaffolds are not superior to uncoated scaffolds in terms of bone formation. The increase in the mean values of the measured parameters after BSP coating is not statistically significant. Bone thickness as well as total bone volume was significantly higher in both scaffold groups in comparison to the negative control. Histological stainings showed a more extensive coverage and infiltration of BSP-coated scaffolds with new bone when matched with uncoated scaffolds. The immunohistological detection of vWF, PECAM-1, and OPN displayed the formation of blood vessels in scaffolds that appeared to be more pronounced in the BSP group. Particularly in scaffolds with BSP coating, OPN-positive osteoblastic cells flocked in multilayers to the newly formed osteoid.

These findings are consistent with our previous in vitro experiments, where we detected increased osteoblastic viability, morphological hOB differentiation, and the spreading of primary human osteoblasts on BSP-coated CPC scaffolds [13]. Graf et al. reported on the increased attraction and attachment of human bone-derived cells and an enhanced transforming growth factor beta (TGF-ß)-expression on BSP-coated hydroxyapatite-based titanium implants in vitro [34]. In another in vitro study, Hilbig et al. found that pre-coating with BSP improved the proliferation of human osteoblasts on a smooth glimmer more than on titanium with hydroxyapatite ceramic [35]. In vivo experiments investigating the effect of BSP-coated CPC scaffolds on bone healing are scarce. BSP coating did not have an apparent effect on bone formation following the ectopic implantation of β-tricalcium phosphate scaffolds in nude mice [36]. However, when collagen-binding (CB) peptides derived from BSP were applied to a hydroxyl apatite scaffold, bone growth was significantly stimulated in an in vivo rabbit calvarial defect within two weeks [37]. Chan et al. used BSP-coated polymer networks instead of CPC scaffolds and found enhanced attachment and spreading, but no effect on the proliferation of MC3T3-E1 cells in vitro [38]. BSP–collagen scaffolds stimulated osteoblast differentiation, bone growth, and vascularization in vivo in a rat calvarial defect model [39]. In a rat model, BSP–collagen scaffolds induced early mineral deposition and resulted in more newly formed bone in comparison to collagen scaffolds in 8-mm calvarial bone defects [40].

A possible limitation of our study was that an eight-week-period from implantation till µCT assessment may be too long for investigating the differences between the scaffolds’ potential for osteoconduction and osteoinduction in the early phase of bone regeneration. Since uncoated scaffolds are shown to have noticeable bone ingrowth after eight weeks, a shorter observation period might have allowed detecting a possible increase in the bone formation rate through BSP coating. The bone formation rate is of main interest: larger scaffolds (as used in large animal models or human beings) could be interpenetrated in a shorter time span, and the weight bearing of extremities could start earlier. Even if there is a positive effect of BSP coating on the new formation of bone, the sample size in our study might have been too small to provide the needed evidence. An anchoring of the scaffold in the calvarial defect (e.g. with fibrin glue or through a conical shape of the scaffold) might have led to a higher rate of bone–implant contacts due to less scaffold mobility, and finally, to less exclusions from analyses resulting in a sample size that is high enough to detect statistically significant differences.

Although our results are in general agreement with previous findings, we were not able to prove the superiority of BSP coating over uncoated scaffolds in the murine calvarial model eight weeks after implantation. We found vessel formation and the formation of new bone in both scaffold groups (BSP coated and uncoated scaffolds). The advantage of BSP coating over uncoated scaffolds may be provided in the early stages after scaffold implantation. Thus, further investigations are required to determine the time dependency of vessel and bone formation early after scaffold implantation.

## 5. Conclusions

In our mouse calvarial defect model, coating CPC scaffolds with BSP showed a tendency towards an increased bone formation in comparison to uncoated CPC scaffolds in bone fractions, BV/TV, and histological sections, but the observed effects were not statistically significant after eight weeks. These parameters differed significantly between both scaffold groups and the negative control. Both BSP-coated and uncoated CPC scaffolds revealed massive bone ingrowth, albeit bone growth in histological stainings was found to be more extensive than in the former. The structure of the newly formed bone resembled that of natural trabecular bone when BSP was applied to the scaffolds. Immunohistological staining presented that vessel formation and OPN+ cell population was pronounced in the BSP group. Our results demonstrate the feasibility of BSP coating, although exceptional effects on bone growth were not detectable. However, further studies could deal with the influence of the scaffolds´ material on the effect of BSP, and should focus on the investigation of spatio-temporal patterns of bone formation and vascular ingrowth in vivo.

## Abbreviations

AB (antibody), BIC (bone-implant contact), BSA (bovine serum albumin), BSP (bone sialoprotein), BV (bone volume), BV/TV (bone volume/total volume), CPC (calcium phosphate cement), DAB (3,3′-diaminobenzidine), ECM (extracellular matrix), HE (haematoxylin eosin), IPA (isopropyl alcohol), MGT (Masson-Goldner-Trichrom), OPN (osteopontin), PBS (phosphate-buffered saline), PECAM-1 (platelet endothelial cell adhesion molecule-1), ROI (region of interest), RP (rapid prototyping), TV (total volume), vWF (von Willebrand factor).
